# Diagnostic value of computed tomography‐based pulmonary artery to aorta ratio measurement in chronic obstructive pulmonary disease with pulmonary hypertension: A systematic review and meta‐analysis

**DOI:** 10.1111/crj.13485

**Published:** 2022-03-14

**Authors:** Xing‐gui Wu, Yu‐jia Shi, Xiao‐hua Wang, Xiao‐wei Yu, Ming‐xia Yang

**Affiliations:** ^1^ Department of Respiratory and Critical Care Medicine The Affiliated Changzhou No. 2 People's Hospital of Nanjing Medical University Changzhou China

**Keywords:** COPD, diagnostic accuracy, pulmonary artery to aorta ratio, pulmonary hypertension

## Abstract

**Objective:**

We conducted a meta‐analysis to systematic assess the diagnostic value of computed tomography (CT)‐based pulmonary artery to aorta (PA:A) ratio measurement in COPD with pulmonary hypertension (COPD‐PH).

**Methods:**

Published studies referring to diagnostic accuracy of PA:A ratio for COPD‐PH were screened out from PubMed, Embase, Web of science, China National Knowledge databases (CNKI), Wan fang databases, and VIP databases. We used bivariate random‐effects model to estimate pooled sensitivity (SEN), specificity (SPE), positive and negative likelihood ratios (PLR and NLR, respectively), and diagnostic odds ratios (DOR). Summary receiver operating characteristic (SROC) curves and area under the curve (AUC) were also calculated to summarize the aggregate diagnostic performance.

**Results:**

Nine eligible studies were included and the pooled SEN was 69% (95% CI: 59 ~ 78), SPE was 85% (95% CI: 77 ~ 90), PLR was 4.5 (95% CI: 2.8 ~ 7.5), and NLR was 0.36 (95% CI: 0.26 ~ 0.51), respectively. DOR reached 13.00 (95% CI: 6.00 ~ 28.00), and value of AUC was 0.84 (95% CI: 0.81 ~ 0.87). Subgroup analysis indicated that when the value of PA:A ratio was equal or greater than one (PA/A ≥ 1), the combined SEN, SPE, AUC, and DOR was 69%, 89%, 0.90, and 19.65, respectively.

**Conclusions:**

PA:A ratio is helpful for appraisal of COPD‐PH, and PA/A ≥ 1 possessed prominent diagnostic accuracy.

## Graphical abstract

We conducted a systematic review and meta‐analysis of available evidence on the accuracy of PA: A ratio for the diagnosis of pulmonary hypertension with COPD(COPD‐PH) and proved that CT‐based PA:A ratio measurement was a distinctively non‐invasive biomarker to complement diagnosis of COPD‐PH in the aspect of accuracy and specificity. Meanwhile, PA/A ≥ 1 may be an appropriate select.

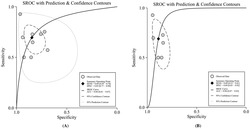

## 1 INTRODUCTION

As a common chronic respiratory disease, chronic obstructive pulmonary disease (COPD) presents a condition with sophisticated clinical manifestations and pathophysiological features, which is featured with persistent respiratory symptoms and irreversible airflow limitation.^1^

In addition to causing noteworthy economic and social burden, it was estimated that COPD would become the third leading cause of deaths globally in 2030.^2^

The histological abnormality of COPD is the result of the combined action of many factors, such as small airway lesion, pulmonary parenchymal destruction, and pulmonary vascular reconsitution.^3^ Cardiovascular comorbidities have a greater prevalence in COPD and associates with increased acute exacerbation and mortality,^4^ which usually cannot be detected or estimated using conventional pulmonary function tests. Pulmonary hypertension (PH) occurs frequently across different stages of COPD,^5^ especially in patients with advanced stage, and the prevalence rate of PH with COPD (COPD‐PH) has been reported varying from 20% to 90%.^6^ PH is defined a mean pulmonary arterial pressure (mPAP) equal or higher than 25 mmHg at rest and measured by right heart catheterization (RHC).^7^, ^8^ However, as an invasive assessment method with numerous adverse complications, in fact, RHC cannot be widely popularized in clinical application. In contrast, comprehensive echocardiography is essential for screening and initial noninvasive assessment of PH.^9^

Computed tomography (CT), a routine noninvasive check inspection device, has become a valuable technique for the assessment of morphological characteristics in COPD, such as emphysema, bronchial lesions, and pulmonary vessels, respectively.^10^, ^11^, ^12^ A large prospective study showed that CT‐based pulmonary artery to aorta (PA:A) ratio on CT scan would be a remarkable parameter to predict the risk of COPD exacerbation and hospitalization.^13^ Furthermore, the PA:A ratio has also been shown to outperform echocardiogram at predicting PH in patients with severe COPD.^14^

To better accurately identify and select a distinctively characteristic sign of COPD‐PH, we undertook this systematic review and meta‐analysis to summarize the subsistent evidence on the accuracy of PA:A ratio for diagnosis of COPD‐PH.

## 2 METHODS

### 2.1 Publications selection

We searched PubMed, Embase, and Web of science with the search strategy of medical subject heading (MeSH) terms together with Entry Terms, which included “Chronic obstructive pulmonary disease,” “COPD,” “Computed Tomography,” “CT,” “pulmonary hypertension,” and “PH.” In the same way, databases of China National Knowledge Internet (CNKI), VIP, and Wan fang databases were searched as well. There were no restrictions in the date of publication and language.

The qualified study must involve in diagnostic accuracy of PA:A ratio for COPD‐PH, and all patients conformed to the diagnostic criteria of Global Initiative for Chronic Obstructive Lung Disease (GOLD) guideline, and the PH was diagnosed by RHC or echocardiography. The sensitivity (SEN) and specificity (SPE), meanwhile, were reported in the original research, or true negative (TN), true positive (TP), false negative (FN) and false positive (FP) can be calculated from the reported information. We excluded repetitive studies, animal research, reviews, case reporting, and literature with insufficient data. Two investigators (WXG and SYJ) independently retrieved eligible literature to preliminary analysis and reached an agreement.

### 2.2 Data extraction and quality assessment

The contents of the extraction information include first author, publication year, country, sample size, diagnostic apparatus, cut‐off value of PA:A ratio, and area under the receiver operating characteristic curve (AUC), SEN, and SPE of diagnostic accuracy. All eligible research was assessed with Quality Assessment of Diagnostic Accuracy Studies‐2 (QUADAS‐2)^15^ criteria, which contained patient selection, index test, reference standard, flow, and timing. The included articles were evaluated as “low,” “high,” or “unclear” independently by two researchists and crosschecked.

### 2.3 Statistical analysis and data synthesis

The pooled estimates of SEN, SPE, and diagnostic odds ratio (DOR) were used to evaluate diagnostic value of PA:A ratio, and rational summary receiver operator characteristic (SROC) curve, AUC was assessed for diagnostic accuracy. Heterogeneity was quantified using Cochran's *Q* test, and *P* < 0.1 indicated as conspicuous heterogeneity. Commonly, the primary source of heterogeneity in test accuracy study is threshold effect. We therefore computed spearman correlation coefficient to reveal the threshold effect, if the coefficient showed strong negative correlation and *P* < 0.05, indicating the existence of threshold effect.^16^ Deek's funnel plot asymmetry tests were conducted to assess publication bias. All data analyses were performed using STATA version 14 and Meta‐Disc version 1.4 statistical software.

## 3 RESULTS

### 3.1 Characteristics of included studies and quality assessment

**FIGURE 1:**
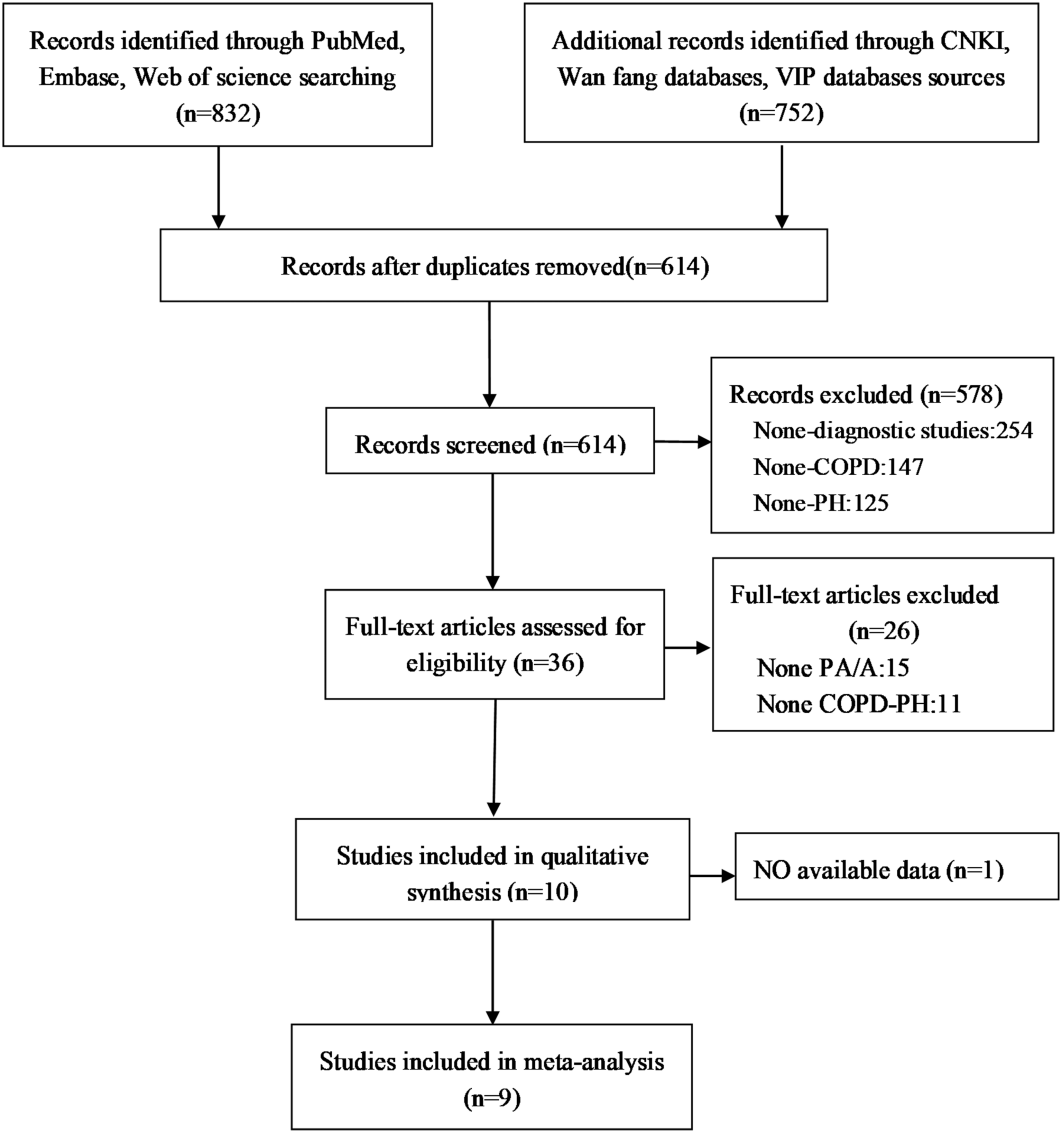
Following diagram illustrating study selection

**FIGURE 2:**
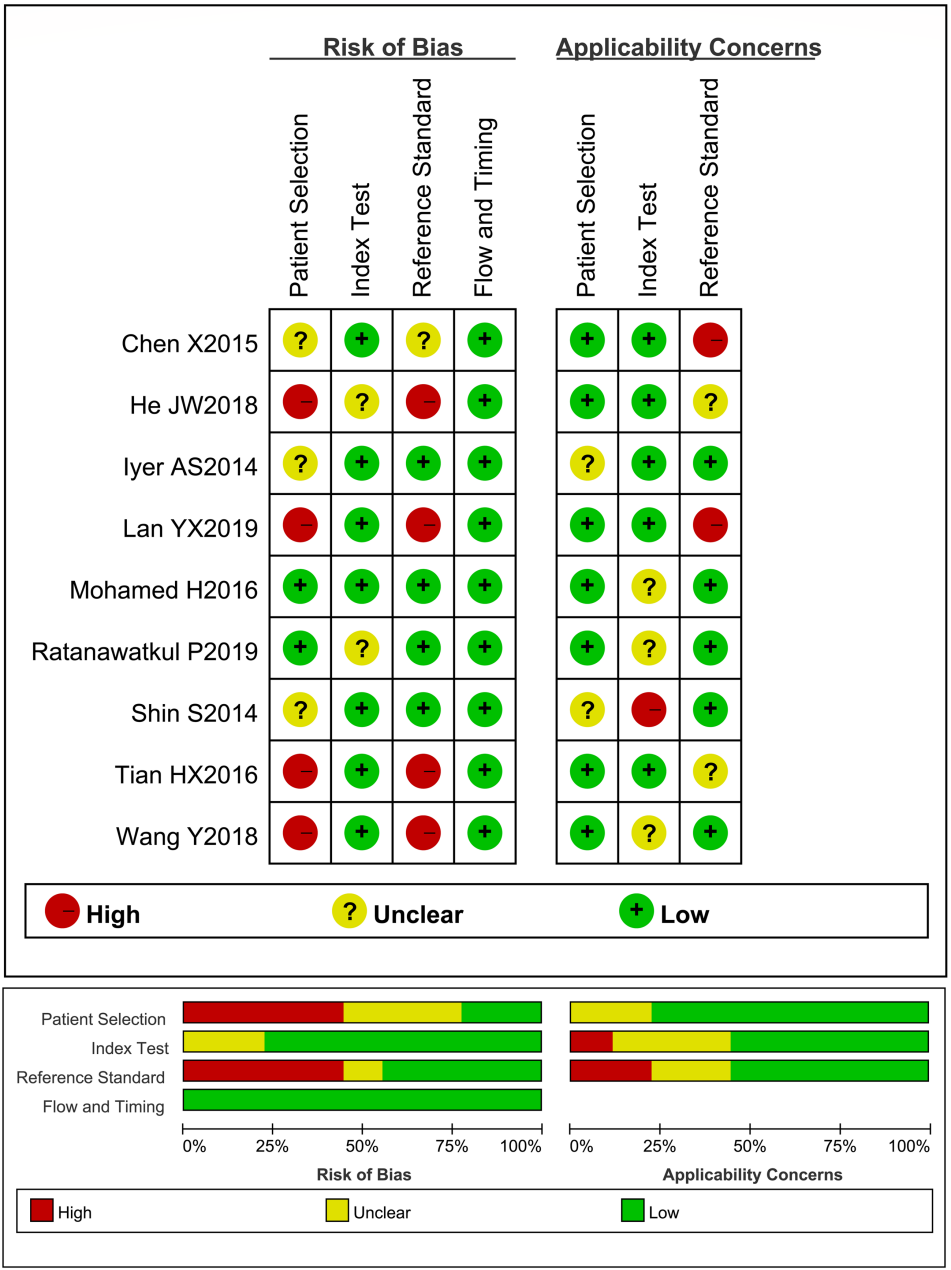
Methodological quality summary of individual studies

A total of 1584 manuscripts were retrieved from published research. After eliminating of duplicates and reading of the titles/abstracts, we agreed on 36 articles meeting inclusive criteria to further browse the full text. Ultimately, nine retrospective studies^14^, ^17^, ^18^, ^19^, ^20^, ^21^, ^22^, ^23^, ^24^ were taken into our review for further meta‐analysis (Figure 1), including 1238 COPD participants, of whom 548 patients were diagnosed as COPD‐PH. The patients with COPD‐PH in five studies were definite diagnosed according to RHC and the cut‐off value of CT‐based PA: A ratio was greater than or equal to one in six research (Table 1). Most of the studies had relatively low bias; however, there were problems in case selection and methodologic limitations, mainly due to unclear information, and the overall quality assessment results were shown in Figure 2.

**TABLE 1:** Clinical characteristics of included studies

Author	year	Country	Simple size		Golden standard	Technique	Cut‐off value of PA:A ratio	Grade of COPD	TP	FP	FN	TN
			COPD‐PH	COPD								
Shin et al.^18^	2014	America	38	27	RHC	LDCT	1.00	GOLD 3–4	19	2	19	25
Iyer et al.^14^	2014	America	22	38	RHC	LDCT	1.00	GOLD 3–4	16	6	6	32
Chen et al.^19^	2015	China	56	165	ECG	HRCT	0.86	NR	32	51	24	114
Mohamed et al.^20^	2016	Netherland	30	62	RHC	HRCT	1.00	GOLD 3–4	15	9	15	53
Wang et al.^21^	2018	China	37	56	RHC	LDCT	1.06	NR	24	9	13	47
Tian and Wang^24^	2016	China	55	55	ECG	HRCT	1.00	NR	51	2	4	53
He et al.^22^	2018	China	43	49	ECG	LDCT	1.00	GOLD 1–2	30	3	13	46
Lan^23^	2019	China	174	195	ECG	LDCT	0.95	GOLD 1–2	127	47	47	148
Ratanawatkul et al.^17^	2020	America	93	43	RHC	HRCT	0.90	NR	70	12	23	31

Abbreviations: COPD, chronic obstructive pulmonary disease; ECG, echocardiography; FN, false negative; FP, false positive; GOLD, Global Initiative for Chronic Obstructive Lung Disease; HRCT, high‐resolution computed tomography; LDCT, low‐dose computed tomography; NR, none report; PA:A, pulmonary artery/aorta ratio; PH, pulmonary hypertension; RCH, right heart catheterization; TN, true negative; TP, true positive.

### 3.2 Diagnostic accuracy of PA/A for COPD‐PH

**FIGURE 3:**
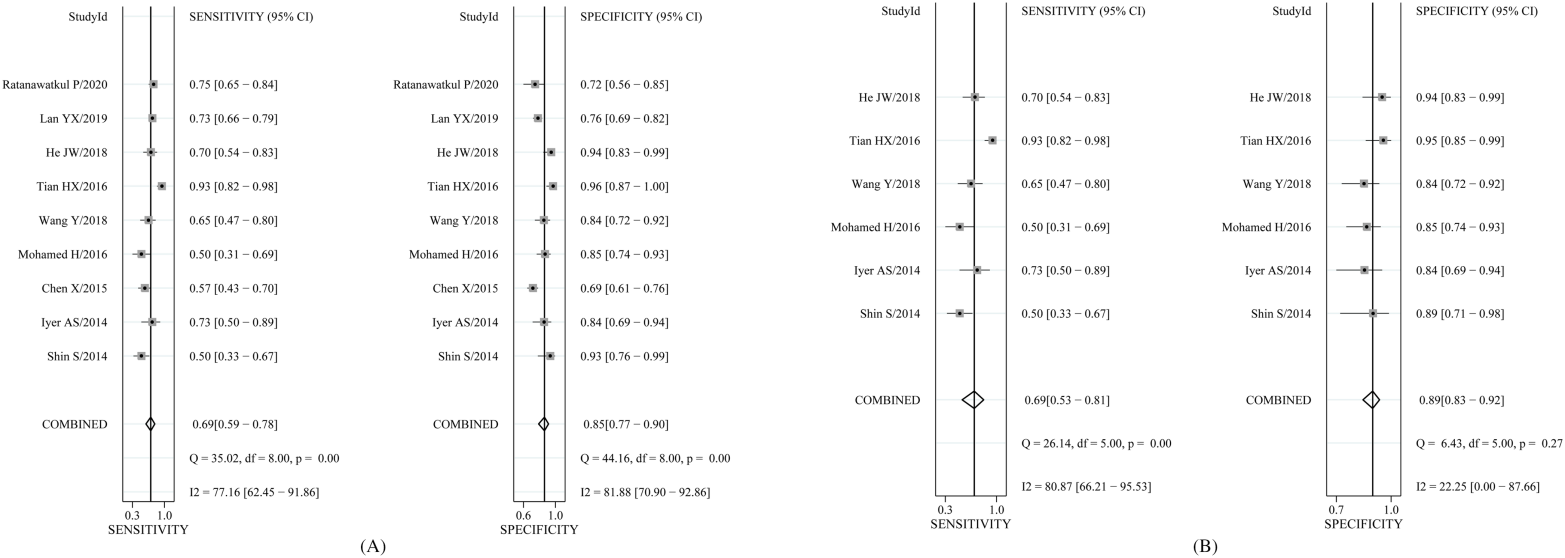
Summary estimates of sensitivity and specificity with corresponding 95% CI for all of the studies (A) and subgroup analysis of PA/A ≥ 1 (B)

**FIGURE 4:**
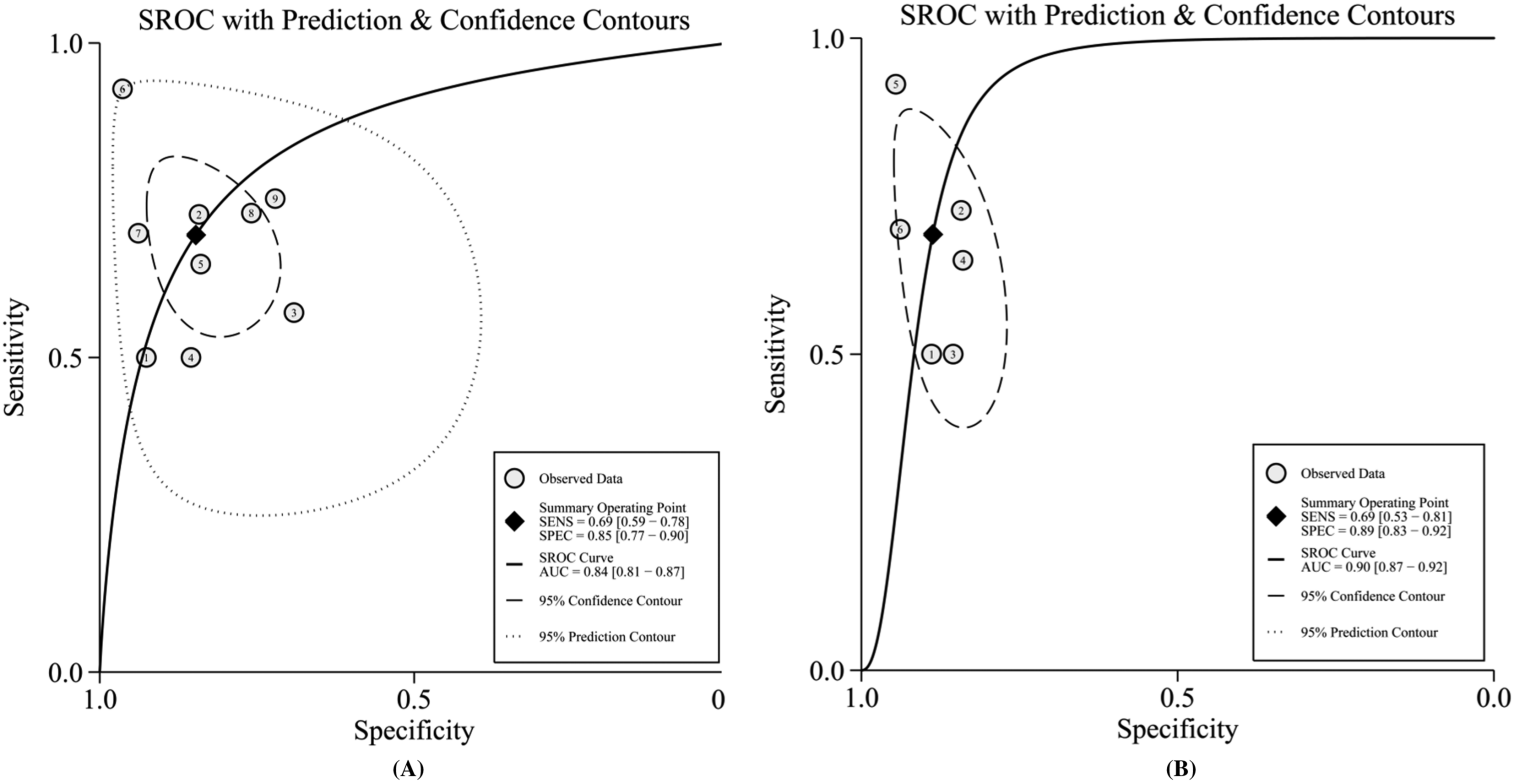
Summary receiver operating characteristic curve (sROC) for all of the studies (A) and subgroup analysis of PA/A ≥ 1 (B)

There was no threshold effect (*r* = −0.02, *P* = 0.95) according to spearman correlation coefficient but significant heterogeneity between different studies (Figure 3). Race, severity of COPD, inspection equipment of CT, golden standard, and the cut‐off value of PA/A were taken into account, and our meta‐regression analysis indicated that heterogeneity existed in the various cut‐off value of PA/A (PA/A ≥ 1 or <1) or other indeterminate covariates. The SEN was 69% (95% CI: 59 ~ 78), and SPE reached 85% (95% CI: 77 ~ 90). The pooled positive likelihood ratio (PLR) was 4.5 (95% CI: 2.8 ~ 7.5), and negative likelihood ratio (NLR) was 0.36 (95% CI: 0.26 ~ 0.51). The pooled DOR was estimated to 13.00 (95% CI: 6.00 ~ 28.00), and the AUC reached 0.84 (95% CI: 0.81 ~ 0.87) (Figure 4A). At the same time, the summary of subgroup analysis was reported in Table 2, and when the value of PA:A ratio was more than or equal to one (PA/A ≥ 1), the combined SEN, SPE, AUC, and DOR was 69%, 89%, 0.90, and 19.65, respectively (Figure 4B).

**TABLE 2:** Diagnostic accuracy summary of subgroup analysis

Diagnostic Index	SEN (95%CI)	SPE (95%CI)	PLR (95%CI)	NLR (95%CI)	DOR (95%CI)	AUC (SE)
Cut‐off value						
PA/A ≥ 1	0.69 (0.50–0.84)	0.89 (0.84–0.93)	6.06 (3.41–10.76)	0.40 (0.23–0.56)	19.65 (7.26–53.16)	0.90 (0.05)
PA/A < 1	0.71 (0.66–0.76)	0.73 (0.68–0.77)	2.47 (1.76–3.46)	0.42 (0.29–0.62)	5.85 (2.93–11.68)	0.80 (0.04)
Golden standard						
RHC	0.65 (0.59–0.72)	0.83 (0.78–0.88)	3.52 (2.59–4.77)	0.45 (0.36–0.57)	8.73 (5.46–13.95)	0.81 (0.03)
ECG	0.73 (0.68–0.78)	0.78 (0.74–0.82)	4.85 (2.18–10.80)	0.31 (0.18–0.55)	18.61 (4.63–74.74)	0.83 (0.18)
Race						
Asian	0.72 (0.67–0.77)	0.78 (0.75–0.82)	4.51 (2.36–8.64)	0.34 (0.22–0.53)	15.55 (5.18–46.64)	0.81 (0.16)
Others	0.66 (0.58–0.72)	0.83 (0.76–0.88)	3.38 (2.39–4.78)	0.46 (0.34–0.61)	8.47 (4.97–14.46)	0.81 (0.04)
Inspection equipment						
HRCT	0.72 (0.66–0.77)	0.77 (0.72–0.82)	3.69 (1.68–8.13)	0.36 (0.19–0.66)	11.92 (2.94–48.31)	0.86 (0.15)
LDCT	0.69 (0.63–0.74)	0.82 (0.77–0.85)	4.32 (2.82–6.63)	0.40 (0.32–0.49)	10.63 (7.08–15.96)	0.80 (0.03)

Abbreviations: AUC, area under the curve; DOR, diagnostic odds ratios; ECG, echocardiography; HRCT, high‐resolution computed tomography; LDCT, low‐dose computed tomography; NLR, negative likelihood ratios; PA/A, pulmonary artery/aorta ratio; PLR, positive likelihood ratios; RCH, right heart catheterization; SEN, pooled sensitivity; SPE, specificity.

### 3.3 Publication bias

**FIGURE 5:**
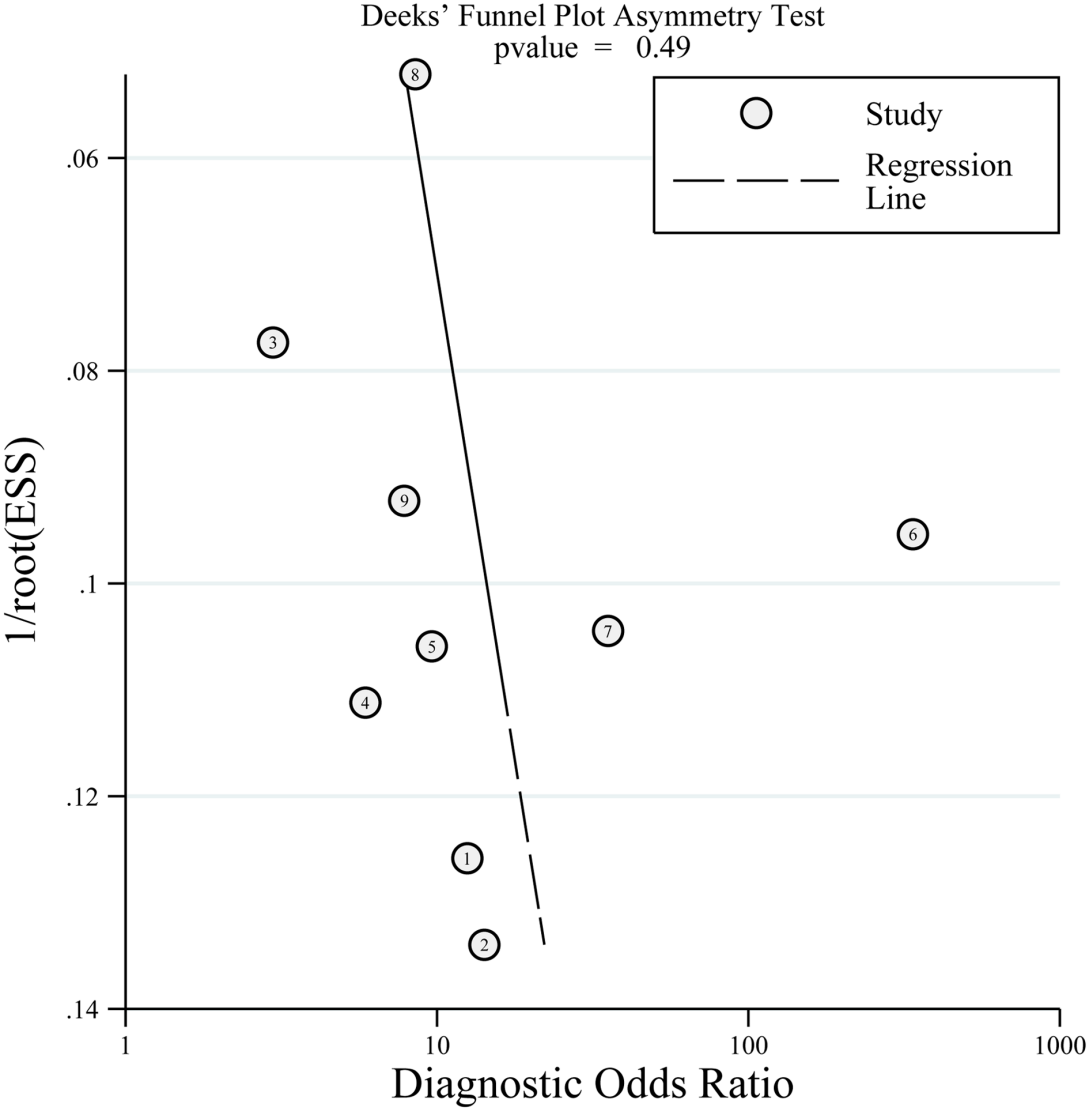
Funnel plot for evaluation of publication bias in all studies

The Deek's funnel plot asymmetry test was not statistically significant (*P* = 0.49) with a symmetrical funnel plot (Figure 5).

## 4 DISCUSSION

Our meta‐analysis of diagnostic accuracy to summarize related research evidence and proved that a CT‐based PA/A ≥ 1 presents a vital role in the diagnosis of COPD‐PH in the aspect of accuracy and SPE, which would be a valuable imaging marker to further assessment of COPD.

COPD possesses complex pathophysiological features, pulmonary ventilation dysfunction and hypoxia due to emphysema and airway inflammation can further trigger pulmonary vasoconstriction and remodeling, pulmonary vascular bed destruction, and increase pulmonary vascular resistance, resulting in PH.^25^, ^26^ In addition, certain global perspective research has revealed that about 30–70% of mild and moderate COPD patients accompanied PH, and a higher prevalence emerged in advanced COPD.^27^, ^28^ Therefore, early recognition of PH in patient with COPD is critical to the comprehensive management of COPD. In recent years, quite a number of studies have found that quantitative measurements of the PA:A ratio on pulmonary CT images in patients with COPD were helpful in identification of PH and closely associated with higher mortality, increased risk on exacerbations, and quality of life.^29^, ^30^, ^31^, ^32^ All these studies have also manifested a feasible noninvasive clinical tool for management of COPD‐PH.

In fact, a meta‐analysis had indicated that PA:A ratio suggested a moderate overall accuracy to distinguish PH in average patients, and value of AUC was 0.84.^33^ However, we focused attention on the population of COPD and incorporated the existing research and proved that PA:A ratio indicated a better diagnostic effect of PH in patients with COPD in terms of the high value of AUC, especially when PA/A ≥ 1, the value reached 0.90. The random effects method, moreover, was performed to reckon the amalgamative SEN and SPE of SROC due to non‐negligible heterogeneity, and PA:A ratio was related to a high SPE, medium sensitivities in diagnosing of COPD‐PH, signifying a relative lower rate of misdiagnosis and high missed diagnosis. DOR is another indicator to evaluate the authenticity and reliability of diagnostic test, which reflects the degree of connection between the diagnostic index and the disease, and higher values mean better discriminatory test performance. The pooled DOR in our meta‐analysis was estimated as 19.65, indicating that PA:A ratio should be feasible index to the diagnosis of COPD‐PH.

The heterogeneity in these studies was explored through the meta‐regression analysis of possible influencing factors, such as Race, cut‐off value of PA:A ratio, severity of COPD, inspection equipment of CT, and golden standard; we found that the value of cut‐off may be a source of heterogeneity, especially when the PA/A value is bounded by one. At present, the optimum value of PA:A to discern COPD‐PH has not been affirmed; the cut‐off value ranges from 0.86 to 1.06 among our eligible studies. On the strength of our review, a higher SPE and diagnostic accuracy displayed when the value PA:A ratio was equal or greater than one, which highlighted a good discriminatory ability to COPD‐PH. For that matter, large prospective study should be conducted to further verification, and further research also showed a considerable screening value of PA:A ratio in patients with COPD‐PH whether using echocardiography or RHC as the gold standard. Meanwhile, in terms of diagnostic authenticity and SEN, the high‐resolution computed tomography (HRCT) examination present a more advantage from our subgroup analysis.

As a systematic review and meta‐analysis, we reviewed the previous research results and obtained valuable evidence‐based medicine testimony; however, limitations should not be neglected. First, a relatively small final included studies and existing of select bias in sectional studies that influenced the accuracy of the results. Moreover, there also was indecipherable heterogeneity among studies even though random‐effects model was used to amalgamate effect size.

## 5 CONCLUSIONS

In conclusion, CT‐based PA:A ratio measurement proved a noninvasive confirmatory test to complement diagnosis of COPD‐PH, and PA/A ≥ 1 may be an appropriate select.

## CONFLICT OF INTEREST

The authors have no conflicts of interest to declare.

## ETHICS STATEMENT

The ethical committee approval was not required at our institution because our research is a systematic review and meta‐analysis, and all analyses were based on previous published studies.

## AUTHOR CONTRIBUTIONS

Xing‐gui Wu, Yu‐jia Shi, Xiao‐hua Wang, Xiao‐wei Yu performed the conceptualization. Xing‐gui Wu, Yu‐jia Shi, Xiao‐hua Wang, Ming‐xia Yang did the writing, original draft, systematic review, and meta‐analysis. Xiao‐hua Wang and Xiao‐wei Yu did the review and editing.

## Data Availability

The data that support the findings of this study are available from the corresponding author upon reasonable request.
